# Inhibition of lncRNA NFIA-AS1 Alleviates Abnormal Proliferation and Inflammation of Vascular Smooth Muscle Cells in Atherosclerosis by Regulating miR-125a-3p/AKT1 Axis

**DOI:** 10.1155/2023/8437898

**Published:** 2023-04-04

**Authors:** Yi Zhu, Xiaofeng Tian, Yan Wang, Chengxiang Wang, Naiquan Yang, Lianghong Ying, Hongyan Niu

**Affiliations:** ^1^Department of Cardio-Thoracic Surgery, The Affiliated Huai'an Hospital of Xuzhou Medical University and The Second People's Hospital of Huai'an, No. 60, Huaihai Road (South), Huaian 223002, China; ^2^Internal Medicine-Cardiovascular Department, The Affiliated Huai'an Hospital of Xuzhou Medical University and The Second People's Hospital of Huai'an, No. 60, Huaihai Road (South), Huaian 223002, China; ^3^Clinical Laboratory, The Affiliated Huai'an Hospital of Xuzhou Medical University and The Second People's Hospital of Huai'an, No. 60, Huaihai Road (South), Huaian 223002, China

## Abstract

Vascular smooth muscle cells (VSMCs) are critical elements of the vascular wall and play a crucial role in the genesis and development of atherosclerosis (AS). Increasingly, studies have indicated that long noncoding RNAs (lncRNAs) regulate VSMC proliferation, apoptosis, and other biological processes. Nevertheless, the role of lncRNA NFIA-AS1 (hereinafter referred to as NFIA-AS1) in VSMCs and AS remains unclear. Quantitative real-time PCR (qRT-PCR) was performed to analyze the messenger RNA (mRNA) levels of NFIA-AS1 and miR-125a-3p. CCK-8 and EdU staining were performed to detect VSMC proliferation. VSMC apoptosis was evaluated by flow cytometry. The expression of various proteins was detected using western blotting. The levels of inflammatory cytokines secreted by VSMCs were measured by enzyme linked immunosorbent assay (ELISA). The binding sites of NFIA-AS1 and miR-125a-3p, as well as miR-125a-3p and AKT1, were analyzed using bioinformatics methods and validated using a luciferase reporter assay. The function of NFIA-AS1/miR-125a-3p/AKT1 in VSMCs was clarified through loss- and gain-of-functional experiments. We confirmed that NFIA-AS1 was highly expressed in AS tissues and VSMCs induced by oxidized low-density lipoprotein (Ox-LDL). Knockdown of NFIA-AS1 restrained the exceptional growth of Ox-LDL-induced VSMCs, promoted their apoptosis, and decreased the secretion of inflammatory factors and expression of adhesion factors. In addition, NFIA-AS1 regulated the proliferation, apoptosis, and inflammatory response of VSMCs through the miR-125a-3p/AKT1 axis, suggesting that NFIA-AS1 may be a potential therapeutic target for AS.

## 1. Introduction

Atherosclerosis (AS) is the main cause of a variety of ischemic cardiovascular diseases, such as ischemic stroke, coronary heart disease, and thrombosis, which critically endanger health and life [1]. The formation of plaques includes lipid infiltration, inflammatory cell adhesion, migration and propagation of smooth muscle cells, formation of the extracellular matrix, and intraplaque hemorrhage [2]. Studies have found that excessive growth and aberrant movement of vascular smooth muscle cells (VSMCs) are crucial factors in the occurrence of AS [3]. Maintaining the dynamic balance between the proliferation and apoptosis of VSMCs and retarding their aberrant migration are important for maintaining the normal functions of blood vessels, which is also a promising strategy for the treatment of AS [4].

Long noncoding RNAs (lncRNAs) are a type of noncoding RNA with a length greater than 200 nucleotides, and are located in the nucleus or cytoplasm [5]. Through transcriptional and post-transcriptional regulation, lncRNAs participate in the regulation of various physiological processes, such as the cell cycle, proliferation, and apoptosis [6, 7]. lncRNAs can compete to bind to miRNA response elements through a competitive endogenous RNA (ceRNA) mechanism, thereby regulating the level and function of target messenger RNA (mRNA) [8]. Recently, lncRNAs have been found to affect the process of AS by regulating vascular wall function, lipid metabolism, inflammatory responses, and immune response [9–12]. Several studies have shown that lncRNAs are significantly involved in the regulation of VSMCs [13]. lncRNA 430945 was reported to promote the propagation and migration of VSMCs via the ROR/RhoA pathway [14]. Bai et al. found that lncRNA-MEG3 was downregulated in AS, sponging miR-26a through a ceRNA mechanism, and regulating the balance between growth and apoptosis of VSMCs by controlling the miR-26a/Smad1 axis [15]. Liu et al. confirmed that downregulation of lncRNA AK094457 inhibits the abnormal proliferation, migration, reactive oxygen species (ROS) production, and inflammation of VSMCs induced by oxidized low-density lipoprotein (Ox-LDL) [16].

lncRNA NFIA-AS1 (hereinafter referred to as NFIA-AS1) is a novel lncRNA located on chromosome 1p31.3 [17], also known as RP5-833A20.1 [18], and is an intronic antisense lncRNA of the NFIA gene [19]. NFIA inhibits the formation of AS plaques by enhancing reverse cholesterol transport [20, 21]. Using microarray analysis, Hu et al. found that NFIA-AS1 was upregulated in THP-1-derived foam cells and inhibited the expression of NFIA by upregulating miR-382-5p [22]. NFIA-AS1 was induced by high levels of oxidized and acetylated LDL, and was confirmed to regulate cholesterol homeostasis and inflammatory response in AS via the miR-382-5p/NFIA axis [22]. However, there is no research on the regulation of NFIA-AS1 in VSMCs in AS. In the present study, we found increased expression of the NFIA-AS1 in both AS tissues and Ox-LDL-induced VSMCs. Knockdown (KD) of NFIA-AS1 restrained the Ox-LDL-induced multiplication of VSMCs, promoted their apoptosis, and reduced the secretion of inflammatory factors and expression of adhesion factors. Furthermore, we confirmed that NFIA-AS1 regulates the proliferation, apoptosis, and inflammatory response of VSMCs through the miR-125a-3p/AKT1 axis.

## 2. Methods

### 2.1. Clinical Specimens

Thirty patients and 30 healthy volunteers admitted to the Affiliated Huai'an Hospital of Xuzhou Medical University from May 2020 to May 2021 were included in this study. Informed consent was obtained from all subjects. Venous blood was centrifuged to obtain serum. The experimental protocol was approved by the Affiliated Huai'an Hospital of Xuzhou Medical University.

### 2.2. Cell Culture

Human VSMCs were provided by The Shanghai Cell Bank of Chinese Academy of Sciences. Cells were maintained in dulbecco's modified eagle medium (DMEM) medium supplemented with 10% fetal bovine serum and 1% double-antibody in an incubator at 37°C and 5% CO_2_. All reagents used in the cell culture were purchased from Gibco (Waltham, MA, USA).

### 2.3. Construction of AS Cell Model

The AS cell model was constructed by inducing VSMCs using Ox-LDL [23]. VSMCs with good growth behavior were inoculated in the plate for 24 hours, and then 50 *μ*g/mL Ox-LDL (Sbjbio, Nanjing, China) was injected into the medium and incubated with VSMCs for another 24 hours.

### 2.4. Quantitative real-time PCR (qRT-PCR)

The TRIzol RNA extraction kit (Thermo Fisher Scientific, Waltham, MA, USA) was used to extract RNA from VSMCs and tissues. Briefly, VSMCs or tissues were fully lysed with TRIzol, chloroform was added and centrifuged, the upper water phase was collected and mixed with isopropyl alcohol, centrifuged, and the precipitate was cleaned with 75% ethanol and dried to obtain the required RNA. BlazeTaq One-Step SYBR Green Quantitative real-time PCR (qRT-PCR) Kit (AmyJet, Wuhan, China) and CFX fluorescence qPCR instrument (Bio-Rad Laboratories, Hercules, CA, USA) were used to synthesize complementary DNA (cDNA), construct the qPCR system, and carry out qPCR. All primers were synthesized by GeneCreate (Wuhan, China). All primers used in this study are listed in Table 1. glyceraldehyde-3-phosphate dehydrogenase (GAPDH) and U6 snRNA were selected as internal references for NFIA-AS1, target mRNA, and miR-125a-3p, respectively. The expression of each gene was quantified using the 2^−△△Ct^ method.

### 2.5. Cell Transfection

The synthesized full-length cDNA of NFIA-AS1 was incorporated into the pcDNA3.1 vector (MiaoLingBio, Wuhan, China) for the overexpression of NFIA-AS1, and the empty pcDNA3.1 vector was used as a control. Oligonucleotides, including small interfering RNA (siRNA) against NFIA-AS1 (si-NFIA-AS1) and AKT1 (si-AKT1), mimic and inhibitor of miR-125a-3p, and their non coding sequence (NCS) were synthesized by Thermo Fisher Scientific. The sequences of the oligonucleotides are listed in Table 2. VSMCs were transfected with pcDNA-NFIA-AS1 plasmid or the negative control pcDNA-NC plasmid and oligonucleotides using Lipofectamine 3000 reagent (Thermo Fisher Scientific) following the manufacturer's instructions. The efficiency of cell transfection was examined using qRT-PCR 48 hours after transfection.

### 2.6. Bioinformatics Analysis

The correlation between NFIA-AS1 and AS was analyzed using the LncBook database. The targeting relationship of NFIA-AS1 and miR-125a-3p was predicted by ENCORI. The targets of miR-125a-3p were predicted using miRDB, miRWalk, and TargetScan, and the targets that may be involved in AS were screened using Metascape clustering.

### 2.7. Dual Luciferase Reporter Assay

Wild-type (WT) and mutant (MUT) reporter plasmids of NFIA-AS1 and AKT1 were synthesized by GenePharma (Shanghai, China) and named NFIA-AS1-WT, NFIA-AS1-MUT, AKT1-WT, and AKT1-MUT, respectively. They were co-transfected into VSMCs with the miR-125a-3p mimic or NC mimic. After 48 hours, the fluorescence intensity was measured following the instructions of the Dual-Luciferase Reporter Kit (Promega, Madison, WI, USA), and the relative activity was expressed as the ratio of firefly to renal luciferase activity.

### 2.8. Detection of Cell Viability

The CCK-8 kit (Abcam, Cambridge, UK) was used to evaluate the viability of VSMCs after the different interventions. VSMCs were inoculated in a 96-well plate at 4 × 10^3^ cells, and the same amount of cell-free DMEM was used as a blank control. Each group contained six duplicates. After 0, 24, 48, and 72 hours, the CCK-8 reagent was injected and co-incubated at 37°C for another 2 hours. The OD_450 nm_ value was recorded using a plate reader (Bio-Rad Laboratories), and cell viability was expressed as the Optical Density (OD) value.

### 2.9. Flow Cytometry

Normal and transfected VSMCs (6 × 10^5^ cells) were inoculated into plates. Cells were digested with trypsin (Yuanye Bio-Technology Co. Ltd., Shanghai, China) at 90% confluence. Cells were prepared from a single-cell suspension in cold phosphate belanced solution (PBS). Annexin V-FITC (fluoresceine isothiocyanate) + PI (Propidium Iodide) (Keygen, Changchun, China; 5 *μ*L) was successively added and cells were incubated for 20 minutes in the dark, and the apoptosis rate was detected using a flow cytometer (BD Biosciences, Franklin Lakes, NJ, USA).

### 2.10. EdU Staining Assay

Transfected VSMCs were seeded into 24-well plates and incubated with 50 *μ*M EdU-containing medium for 12 hours. The cells were then washed with PBS three times and fixed with ice-cold methanol for 15 minutes. Cells were stained using an EdU staining kit (Beyotime, Nantong, China) and observed under a fluorescence microscope (Nikon, Tokyo, Japan). Three fields were randomly selected for each well, and the rate of EdU-positive cells was calculated.

### 2.11. Transwell Assay

For invasion detection, the basement membrane of the Transwell chamber (Corning, Corning, NY, USA) was pre-coated with Matrigel (Corning) 1 day before the experiment, and the chambers were placed in 24-well plates. The VSMCs were then inoculated in the upper chamber, and 500 *μ*L of complete DMEM was added to the lower chamber. After 24 hours of culturing, the chamber was removed and stained with 0.1% crystal violet (Aladdin, Shanghai, China). Cells that invaded the bottom were observed under a microscope (Nikon). The process of migration detection was the same as that of invasion detection, except that there was no need to pre-coat the Matrigel in advance.

### 2.12. Western Blot Analysis

VSMCs from each group were collected and lysed on ice using lysis buffer (Solarbio, Beijing, China). The mixture was centrifuged at 12,000 rpm at 4°C for 15 minutes. After discarding the supernatant, the protein concentration was determined using a BCA kit (Merck, Kenilworth, NJ, USA). Equal amounts of protein were used for sodium dodecyl sulfate polyacrylamide gel electrophoresis (SDS-PAGE). The separated proteins were transferred onto a polyvinylidene difluoride (PVDF) membrane, which was then blocked with 5% skimmed milk at 25°C. The membrane was then incubated at 4°C overnight with the following primary antibodies, which were all purchased from Abcam: anti-Bax (ab3191, 1 : 2000), anti-Bcl-2 (ab196495, 1 : 1000), anti-vascular cell adhesion molecule 1 (VCAM-1; ab174279, 1 : 2000), anti- intercellular cell adhesion molecule-1 (ICAM-1; ab109361, 1 : 2000), anti-E-selectin (ab137732, 1 : 2000), anti-GBP-1 (ab22604, 1 : 2000), and anti-glyceraldehyde-3-phosphate dehydrogenase (GAPDH) (ab8245, 1 : 5000). The next day, the membrane was rinsed and incubated with the secondary antibody at 37°C for 30 minutes. Enhanced chemiluminescence (ECL) reagent (Thermo Fisher Scientific) was used to visualize the protein bands and GAPDH was used to normalize the protein levels.

### 2.13. Enzyme linked immunosorbent assay (ELISA)

The supernatant of the transfected VSMCs was collected and the levels of Interleukin-1 (IL-1), IL-1*β*, Tumor Necrosis Factor alpha (TNF-*α)*, and IL-6 were detected using ELISA kits (Sbjbio).

### 2.14. Animal Experiment

A total of 20 six-week-old male ApoE^−/−^ mice were purchased from Cyagen Biosciences (Taicang, China). After adaptive rearing for one week, the mice were randomly divided into four groups: control, model, model + si-NC, and model + si-NFIA-AS1. The control group was fed an ordinary diet, whereas the other groups were fed a high-fat diet. After one month of free feeding, mice in the model + si-NC and model + si-NFIA-AS1 groups were injected with 200 *μ*L of the corresponding adenovirus (1 × 10^10^ pfu/mL) through the tail vein twice a week for 4 weeks. At the end of the experiment, mice in each group were sacrificed under anesthesia, aortas were taken for hematoxylin–eosin (H&E) staining, and venous blood was collected for ELISA and qRT-PCR analysis. The animal experimental protocol was approved by the Animal Ethics Committee of Affiliated Huai'an Hospital of Xuzhou Medical University.

### 2.15. H&E Staining

The aorta was fixed with four paraformaldehyde, and the tissues were embedded in paraffin. The tissues were cut into 4 *μ*M sections. The sections were then stained with H&E (Regal, Wuxi, China) for 3 minutes. The sections were observed and imaged using a microscope (Nikon), and the ImageJ software was used to analyze the images.

### 2.16. Statistical Analysis

All data are presented as mean ± standard deviation from three independent replicates. One-way analysis of variance in the GraphPad Prism (version 8.0; GraphPad Prism Software, San Diego, CA, USA) was applied for the comparison between groups. A *P*-value < 0.05 was considered statistically significant.

## 3. Results

### 3.1. Up-Regulated Expression of NFIA-AS1 Is Associated with AS

**Figure 1:**
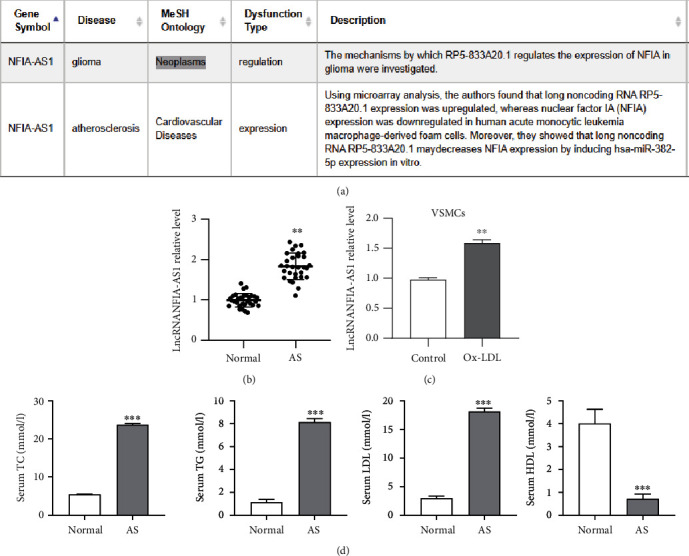
NFIA-AS1 is associated with the progression of AS. (a) Analysis from LncBook database showed that NFIA-AS1 was associated with AS. (b) The expression of NFIA-AS1 in the serum of AS patients and healthy volunteers was detected by qRT-PCR. (c) The expression of NFIA-AS1 in VSMCs and Ox-LDL-induced VSMCs was detected by qRT-PCR. (d) The level of TC, TG, HDL, and LDL in the serum of control and AS patients. ∗∗*P* < 0.01 and ∗∗∗*P* < 0.001.

Analysis using the LncBook database showed that NFIA-AS1 was closely associated with AS (Figure 1(a)). To verify the expression level of NFIA-AS1 in AS, we measured it in the serum of clinical specimens and Ox-LDL-induced VSMCs. The clinical characteristics of the patients are summarized in Table 3. Compared with healthy volunteers, the level of NFIA-AS1 in the serum of patients with AS was significantly elevated (*P* < 0.01; Figure 1(b)). The level of NFIA-AS1 in Ox-LDL-treated VSMCs also increased remarkably (*P* < 0.01; Figure 1(c)). In addition, to further clarify the correlation between NFIA-AS1 and AS, the serum lipid indexes of clinical samples were detected. Compared with the control group, the serum total cholesterol (TC), total triglyceride (TG), and LDL levels in AS patients were significantly increased, whereas the high-density lipoprotein (HDL) content was significantly decreased (*P* < 0.001; Figure 1(d)).

### 3.2. NFIA-AS1 KD Inhibits the Proliferation, Migration, and Invasion of Ox-LDL-Induced VSMCs and Promotes Apoptosis

**Figure 2:**
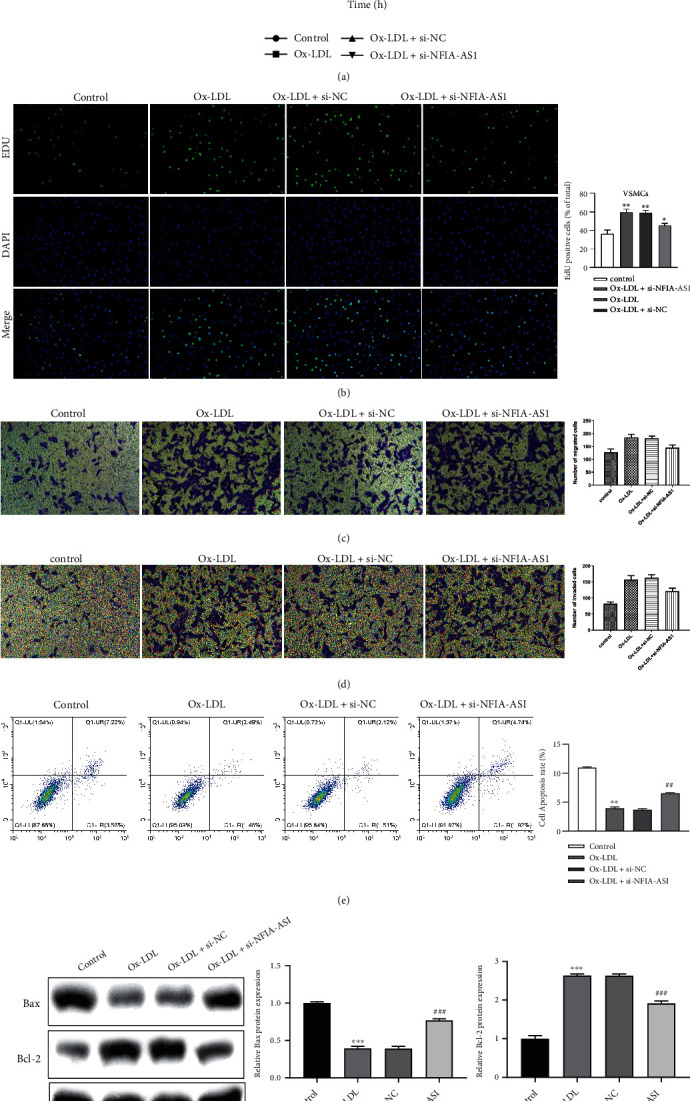
KD of NFIA-AS1 inhibits the proliferation, migration, and invasion of Ox-LDL-induced VSMCs and promotes apoptosis. (a) Cell viability of VSMCs in control, Ox-LDL, Ox-LDL + si-NC, and Ox-LDL + si-NFIA-AS1 groups at 0, 24, 48, and 72 hours were detected by CCK-8 assay. (b) VSMCs in these four groups were stained with EdU. The nucleus was stained with 2-(4-Amidinophenyl)-6-indolecarbamidine dihydrochloride (DAPI). The proportion of EdU-positive cells was counted. Scale bar = 50 *μ*m. The migration (c) and invasion (d) capacity of VSMCs was assessed by Transwell assay. Scale bar = 50 *μ*m. (e) VSMCs were stained with PI and the cell apoptosis was evaluated by flow cytometry. (f) The expression of apoptosis-related proteins Bax and Bcl-2 was determined by western blot. GAPDH was the internal reference. ∗*P* < 0.05, ∗∗*P* < 0.01, and ∗∗∗*P* < 0.001*versus* control group. ^#^*P* < 0.05 and ^##^*P* < 0.01*versus* Ox-LDL + si-NC group.

To explore the function of NFIA-AS1 in AS, we knocked down NFIA-AS1 in Ox-LDL-induced VSMCs. Compared with the Ox-LDL and Ox-LDL + si-NC groups, the cell viability of the Ox-LDL + si-NFIA-AS1 group was markedly decreased at 72 hours (Figure 2(a)). EdU staining analysis showed a similar result; the percentage of EdU-positive cells in the Ox-LDL + si-NFIA-AS1 group was lower than that in the Ox-LDL and Ox-LDL + si-NC groups (Figure 2(b)). In addition, the migration and invasion capacity of VSMCs was weakened in the Ox-LDL + si-NFIA-AS1 group compared with the Ox-LDL and Ox-LDL + si-NC groups (Figures 2(c) and 2(d)). Moreover, the apoptosis ratio of the Ox-LDL + si-NFIA-AS1 group was 6.66%, which was higher than that of the Ox-LDL (3.97%) and Ox-LDL + si-NC (3.63%) groups (Figure 2(e)), indicating that NFIA-AS1 KD accelerated the apoptosis of VSMCs induced by Ox-LDL. This was verified by the levels of the apoptosis-related proteins Bax and Bcl-2 (Figure 2(f)).

### 3.3. NFIA-AS1 KD Alleviates Inflammation in Ox-LDL-Induced VSMCs

**Figure 3:**
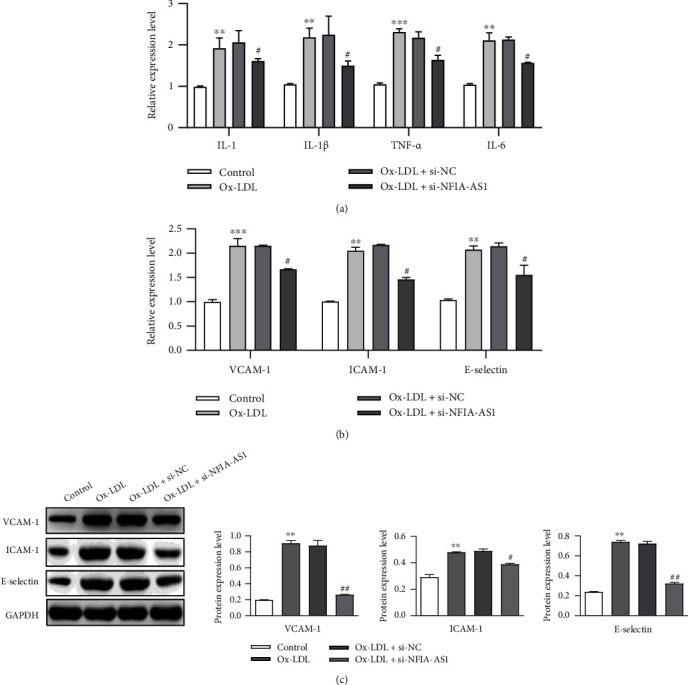
KD of NFIA-AS1 reduces inflammation in Ox-LDL-induced VSMCs. (a) The relative expression levels of inflammatory cytokines in VSMCs of control, Ox-LDL, Ox-LDL + si-NC, and Ox-LDL + si-NFIA-AS1 groups were detected by qRT-PCR. (b) The mRNA expression of VCAM-1, ICAM-1, and E-selectin was detected by qRT-PCR. (c) The protein expression of VCAM-1, ICAM-1, and E-selectin was detected by western blot. ∗∗*P* < 0.01 and ∗∗∗*P* < 0.001*versus* control group. ^#^*P* < 0.05 and ^##^*P* < 0.01*versus* Ox-LDL + si-NC group.

Because AS is an inflammatory disease, we aimed to ascertain the role of NFIA-AS1 in the inflammatory response to AS. First, we assessed the changes in the levels of inflammatory factors secreted by Ox-LDL-induced VSMCs after NFIA-AS1KD using ELISA. The secretion of IL-1, IL-1*β*, TNF-*α*, and IL-6 by VSMCs in the Ox-LDL + si-NFIA-AS1 group was dramatically reduced compared with that in the Ox-LDL or Ox-LDL + si-NC groups (Figure 3(a)). In addition, the mRNA and protein levels of the three adhesion factors, VCAM-1, ICAM-1), and E-selectin, in Ox-LDL-treated VSMCs decreased due to NFIA-AS1 KD (Figures 3(b) and 3(c)). These findings imply that NFIA-AS1 KD is beneficial for inhibiting Ox-LDL-induced inflammation in VSMCs.

### 3.4. MiR-125a-3p Is the Target of NFIA-AS1

**Figure 4:**
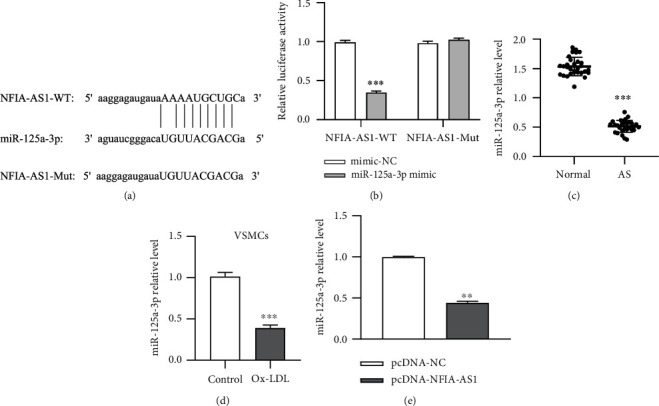
MiR-125a-3p is the target of NFIA-AS1. (a) The binding sites of NFIA-AS1 and miR-125a-3p were predicted by ENCORI. (b) The targeting relationship between NFIA-AS1 and miR-125a-3p was verified by dual-luciferase reporter assay. MiR-NC or miR-125a-3p was co-transfected into VSMCs with luciferase reporter plasmids containing NFIA-AS1-WT or NFIA-AS1-MUT, and the fluorescence intensity was detected. The expression of miR-125a-3p in clinical samples (c) and Ox-LDL-induced VSMCs (d) was detected by qRT-PCR. (e) The expression of miR-125a-3p after transfection with pcDNA-NFIA-AS1 was detected by qRT-PCR. ∗∗*P* < 0.01 and ∗∗∗*P* < 0.001.

The binding sites of miR-125a-3p and NFIA-AS1 were predicted using ENCORI (Figure 4(a)), and their targeting relationships were verified. After transfection with the miR-125a-3p mimic, the relative luciferase activity of the NFIA-AS1-WT group decreased significantly, whereas that of the NFIA-AS1-MUT group almost did not change (Figure 4(b)), indicating that miR-125a-3p was the direct target of NFIA-AS1. Furthermore, miR-125a-3p was downregulated in the serum of AS patients and the supernatant of Ox-LDL-treated VSMCs (Figures 4(c) and 4(d)). In addition, we overexpressed NFIA-AS1 in VSMCs, and qRT-PCR results showed that miR-125a-3p expression was reduced after transfection with pcDNA-NFIA-AS1 (Figure 4(e)). These results confirmed the negative regulatory relationship between miR-125a-3p and NFIA-AS1.

### 3.5. AKT1 Is the Target of miR-125a-3p

**Figure 5:**
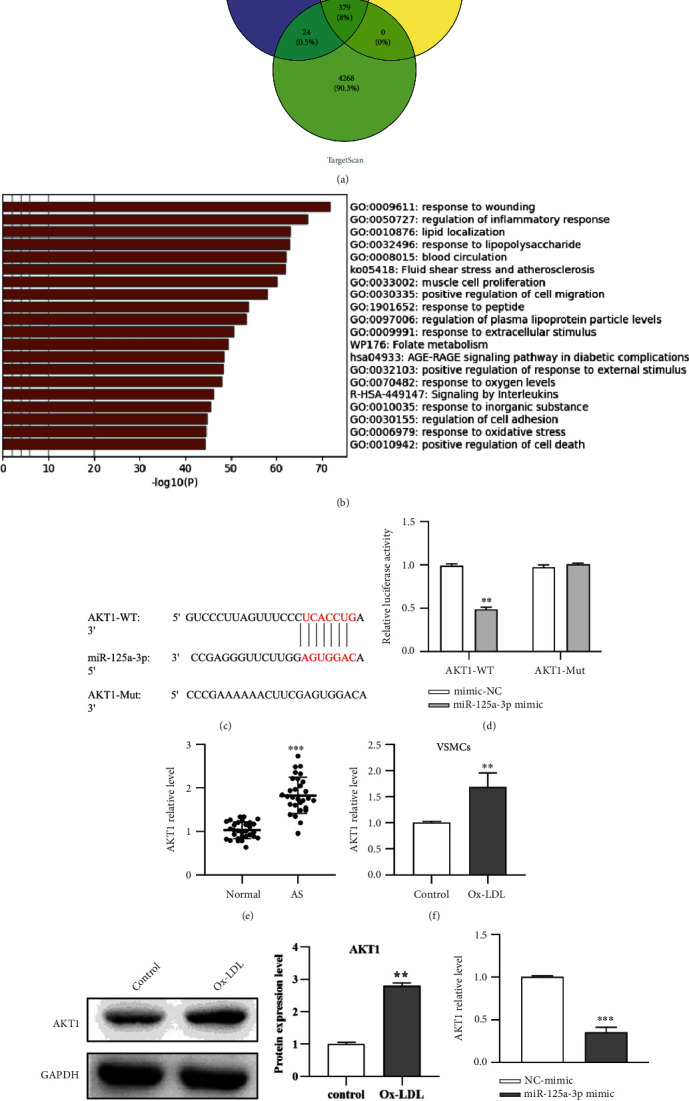
AKT1 is the target of miR-125a-3p. (a) The targets of miR-125a-3p were predicted by miRDB, miRWalk, and TargetScan. (b) Metascape clustering was conducted to screen the targets involved in AS. (c) The binding site of AKT1 and miR-125a-3p was predicted by TargetScan. (d) The targeting relationship between miR-125a-3p and AKT1 was verified by dual-luciferase reporter assay. (e) The expression of AKT1 in clinical samples was detected by qRT-PCR. The expression of mRNA (f) and protein (g) of AKT1 in Ox-LDL-induced VSMCs was detected by qRT-PCR and western blot, respectively. (h) The expression of AKT1 after transfection with miR-125a-3p-mimic was detected by qRT-PCR. ∗∗*P* < 0.01 and ∗∗∗*P* < 0.001.

To screen for the probable targets of miR-125a-3p, miRDB, miRWalk, and TargetScan were used for prediction analysis (Figure 5(a)). Metascape clustering analysis was conducted to screen targets that may be involved in AS (Figure 5(b)), and AKT1 was identified as a candidate. The interaction sites between miR-125a-3p and AKT1 were identified using TargetScan (Figure 5(c)). Their interactive relationship was validated, and it was found that the transfection of the miR-125a-3p mimic dramatically decreased the luciferase activity of VSMCs in the AKT1-WT group (Figure 5(d)). The expression of AKT1 in the serum of AS patients and VSMCs induced by Ox-LDL was upregulated (Figures 5(e), 5(f), and 5(g)). However, after overexpressing miR-125a-3p in VSMCs, AKT1 expression decreased distinctly compared with that in the NC mimic group (Figure 5(h)). These findings illustrate that AKT1 is a downstream target of miR-125a-3p.

### 3.6. NFIA-AS1 Regulates Growth, Apoptosis, and Inflammation of VSMCs in AS through miR-125a-3p/AKT1 Axis

To clarify the mechanism of NFIA-AS1/miR-125a-3p/AKT1 in abnormal proliferation and inflammation of VSMCs in AS, we conducted a series of rescue experiments. Ox-LDL-induced VSMCs were separated into four groups: si-NC + NC-inhibit + si-NC, si-NFIA-AS1 + NC-inhibit + si-NC, si-NFIA-AS1 + miR-125a-3p-inhibit + si-NC, and si-NFIA-AS + miR-125a-3p-inhibit + si-AKT1.

**Figure 6:**
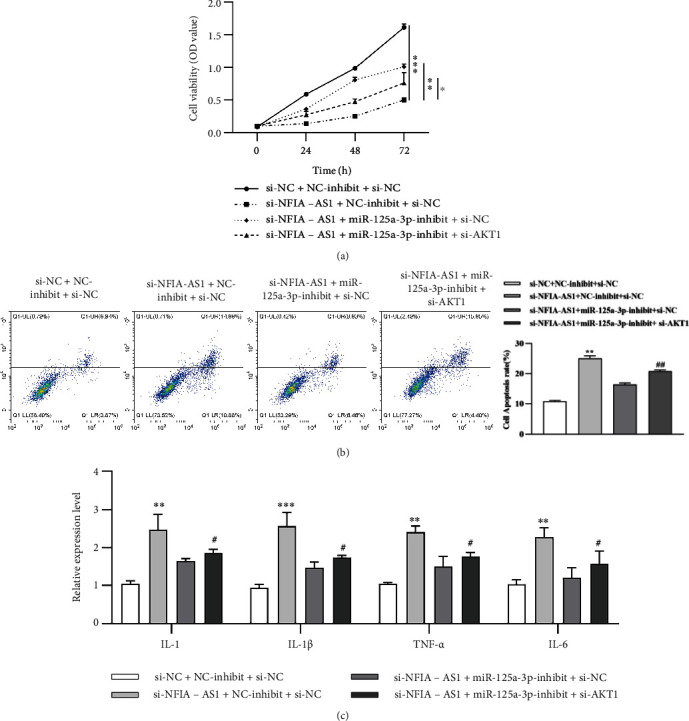
Inhibition of NFIA-AS1 alleviates the proliferation and inflammation of Ox-LDL-induced VSMCs by regulating miR-125a-3p/AKT1 axis. Ox-LDL-induced VSMCs were divided into four groups: si-NC + NC-inhibit + si-NC, si-NFIA-AS1 + NC-inhibit + si-NC, si-NFIA-AS1 + miR-125a-3p-inhibit + si-NC, and si-NFIA-AS1 + miR-125a-3p-inhibit + si-AKT1. (a) Cell viability was detected via CCK-8 assay. ∗*P* < 0.05, ∗∗*P* < 0.01, and ∗∗∗*P* < 0.001. (b) Cells were stained with PI and the apoptosis rate was detected by flow cytometry. (c) The level of inflammatory cytokines was assessed by ELISA. ∗∗*P* < 0.01 and ∗∗∗*P* < 0.001*versus* si-NC + NC-inhibit + si-NC group. ^#^*P* < 0.05 and ^##^*P* < 0.01*versus* si-NFIA-AS1 + miR-125a-3p-inhibit + si-NC group. All results were obtained from three independent experiments.

Compared with the si-NC + NC-inhibited + si-NC group, transfection with si-NFIA-AS1 reduced the viability of VSMCs. Compared with the si-NFIA-AS1 + NC-inhibited + si-NC group, co-transfection with si-NFIA-AS1 and miR-125a-3p-inhibit partially reversed the inhibitory effect of si-NFIA-AS1 and improved cell viability. In addition, co-transfection with si-NFIA-AS1, miR-125a-3p inhibitor, and si-AKT1 inhibited cell viability to a certain extent compared with si-NFIA-AS1 + miR-125a-3p-inhibited + si-NC group, because the function of miR-125a-3p inhibitor was partially reversed by si-AKT1 (Figure 6(a)). Similarly, for cell apoptosis and the secretion of inflammatory cytokines, miR-125a-3p inhibitor reversed the function of si-NFIA-AS1, whereas si-AKT1 weakened the function of the miR-125a-3p inhibitor (Figures 6(b) and 6(c)). These results confirmed that NFIA-AS1 regulates the growth, apoptosis, and inflammation of Ox-LDL-induced VSMCs via the miR-125a-3p/AKT1 axis.

### 3.7. Inhibition of NFIA-AS1 Improves Symptoms of AS Mice

**Figure 7:**
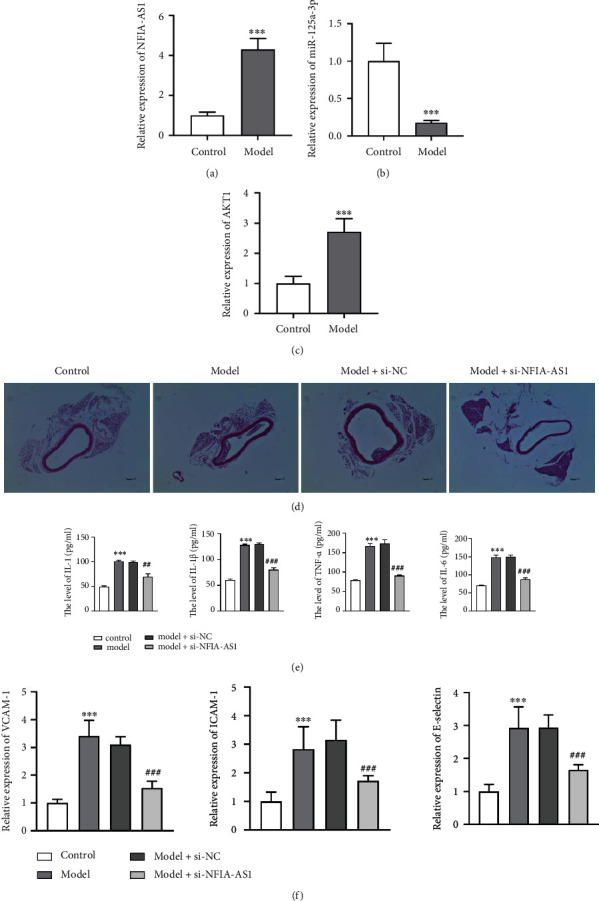
Inhibition of NFIA-AS1 improves the symptoms of AS. The relative expression of NFIA-AS1 (a), miR-125a-3p (b), and AKT1 (c) in control and AS model groups. ∗∗∗*P* < 0.001. (d) H&E staining of aortic vessels of mice in control, model, model + sh-NC, and model + si-NFIA-AS groups. Scale bar = 100 *μ*m. (e) The levels of inflammatory cytokines IL-1, IL-1*β*, TNF-*α*, and IL-6 in the serum of mice were determined by ELISA. (f) The levels of adhesion factors VCAM-1, ICAM-1, and E-selectin in the serum of mice were detected by qRT-PCR. ∗∗∗*P* < 0.001*versus* control group. ^##^*P* < 0.01 and ^###^*P* < 0.001*versus* model + si-NC group.

Next, an AS mouse model was established for *in vivo* study. Consistent with the *in vitro* results, the expression of NFIA-AS1 and AKT1 was significantly increased, whereas the expression of miR-125a-3p was remarkably decreased in the model group (*P* < 0.001; Figures 7(a), 7(b), and 7(c)). H&E staining of the aortic vessels of mice revealed that the vascular lesions in the model + siNFIA-AS1 group were significantly improved (Figure 7(d)). In addition, the levels of inflammatory cytokines IL-1, IL-1*β*, TNF-*α*, and IL-6 in the serum were significantly decreased after NFIA-AS1 KD (*P* < 0.01 and *P* < 0.001; Figure 7(e)). In addition, the levels of the adhesion factors VCAM-1, ICAM-1, and E-selectin were significantly decreased (*P* < 0.001; Figure 7(f)). These results indicated that NFIA-AS1 KD improved the symptoms of AS in mice.

## 4. Discussion

Recent studies have reported the importance of lncRNAs in cardiovascular diseases and that they play an essential regulatory role in the incidence and progression of AS [13, 24]. Therefore, an in-depth study of lncRNAs is of practical significance for understanding the molecular mechanism of AS and provides new directions for the development of novel diagnostic markers and the screening of drug targets.

Here, NFIA-AS1 was elevated in the serum of patients with AS and in the supernatant of Ox-LDL-induced VSMCs. Analysis of the LncBook database also suggested that NFIA-AS1 is related to AS. However, the specific regulatory mechanisms of NFIA-AS1 in AS remain unclear. Therefore, we used Ox-LDL-induced VSMCs as a cell model and knocked down the expression of NFIA-AS1. NFIA-AS1 KD restrained the growth, migration, and invasion of Ox-LDL-induced VSMCs and promoted their apoptosis. Recent studies suggest that AS is an inflammatory disease [25]. Although the factors that cause AS are complex, they eventually lead to chronic inflammation, which manifests as endothelial cell injury, adhesion of monocytes, release of inflammatory factors, and propagation and migration of VSMCs [26, 27]. The adhesion process of monocytes and plaque formation are inseparable from the adhesion factors VCAM-1, ICAM-1, and E-selectin [28]. Here, we demonstrated that NFIA-AS1 KD could reduce the levels of inflammatory and adhesion factors expressed by Ox-LDL-induced VSMCs, suggesting that NFIA-AS1 is involved in regulating the inflammation of AS. Many studies have identified that lncRNAs can function as miRNA sponges to control the expression of their coding genes through the ceRNA mechanism [29, 30]. In the present study, we confirmed the relationship between NFIA-AS1 and miR-125a-3p expression. In addition, miR-125a-3p was inhibited after overexpression of NFIA-AS1, which proved that there was a negative regulatory relationship between NFIA-AS1 and miR-125a-3p.

MiR-125a-3p may be closely related to AS pathogenesis. Xia and Zeng found that miR-125a-3p aggravates Ox-LDL-induced HUVEC injury through BAMBI [31]. Hu et al. confirmed that miR-125a-3p can effectively inhibit the function of VSMCs and the occurrence of vascular stenosis by targeting MAPK1 [32]. Hu et al. showed that miR-125a-3p inhibited the propagation of VSMCs and neointima formation in the carotid arteries of rats [32]. Decreased miR-125a-3p was also found to inhibit the expression of MMP-2 and VEGF and regulate the balance of M1/M2 macrophages, thus lowering the progression of AS plaques [33]. In addition, miR-125a-3p modulates the phenotypic transition of VSMCs by targeting ETS-1 [34]. Nevertheless, the specific regulatory mechanism of miR-125a-3p in AS remains unclear.

AKT1 is an important protein kinase that participates in the regulation of cell growth, apoptosis, and metabolism [35]. Previous studies have indicated that the Akt signaling pathway is involved in AS progression [36]. Inhibition of the expression of AKT1 in VSMCs leads to increased plaques and accelerates VSMC apoptosis [37]. Chen et al. confirmed that miR-155-5p facilitates the growth, migration, and invasion of VSMCs by raising AKT1 in AS [37]. In the current study, we also demonstrated that AKT1 levels were increased in the serum of patients with AS and in VSMCs induced by Ox-LDL. Additionally, the AKT1 levels was negatively regulated by miR-125a-3p. Here, we found that inhibition of NFIA-AS1 reduces cell viability and inflammation of VSMCs in AS via the miR-125a-3p/AKT1 axis.

## 5. Conclusion

To the best of our knowledge, this is the first study to elucidate the involvement of the NFIA-AS1 in the regulation of AS via the miR-125a-3p/AKT1 axis. We confirmed that NFIA-AS1, which is highly expressed in Ox-LDL-induced VSMCs, promotes AKT1 expression by sponging miR-125a-3p, thereby promoting the growth and inflammation of VSMCs. However, the present study had some limitations. We did not verify the effect of the NFIA-AS1/miR-125a-3p/AKT1 axis on VSMCs and the effect on plaque formation and inflammation in the AS mice model. However, this will be explored in future studies. The results of this study provide new targets for early diagnosis and molecular therapy of AS.

## Figures and Tables

**Table 1:** Primers for qRT-PCR.

Gene	Primers	
	Sense	Antisense
lncRNA NFIA-AS1	GCAACTGTTAACTACATAGC	ACGTATCTTGACGACGTACCT
miR-125a-3p	CAGAATGACTTAACGACTAGG	AGACATTGGCATTAACAGCACG
AKT1	CACCTAATCAGTACGCATCA	GCCAACCTTACGACTAGCA
VCAM-1	AGAACATTACGCAGTATTC	GACATAATTGATCAGCATAG
ICAM-1	CGGAATATCGAAGTACGAC	CAGACCTATTCAGCAGAATC
E-selectin	AACGTAGGTTTACGTACAAC	GATCACGACCTGTTACTGACG
GAPDH	AAGCTACAGTACGTTACAGG	CGTACGAAGCAGCTATATGCAG
U6	CGACGTCAGTACGACCTAC	ACGTACGATCGACAGCAATC

**Table 2:** Oligonucleotides for transfection.

	Oligonucleotides	
Name	Sense	Antisense
si-NFIA-AS1	GCAUUACGUUACGAUACGU	CAGACGUAAUUGAUCUGACA
si-AKT1	GCUACCUAGCAUUGAACAGC	AACAGUAGUAUUGACUUGACA
miR-125a-3p mimic	GCGAAGUAGUAAGCUUACG	UUCAGCUUAGAUGCAAUAGA
miR-125a-3p inhibitor	UAGCUUGCAGCUAGCUUUCA	GAUACUUGACGUAGCTAUAGC

**Table 3:** Clinical characteristics of the samples.

Index	AS patients	Healthy control
Male/Female	15/15	15/15
Age	48–72	50–73
Hypertension	13	15
Diabetes	8	9
TC	3.39 ± 0.85	4.26 ± 1.31
TG	1.41 ± 0.33	1.78 ± 0.54
HDL-C	0.99 ± 0.31	1.24 ± 0.53
LDL-C	2.94 ± 0.92	1.77 ± 0.76

## Data Availability

Data supporting this research article are available from the corresponding author or first author on reasonable request.
